# Developing a post-discharge suicide prevention intervention for children and young people: a qualitative study of integrating the lived-experience of young people, their carers, and mental health clinicians

**DOI:** 10.1186/s13034-022-00460-3

**Published:** 2022-03-27

**Authors:** David P. Watling, Megan H. W. Preece, Jacinta Hawgood, Sharyn Bloomfield, Kairi Kõlves

**Affiliations:** 1 Australian Institute for Suicide Research and Prevention, World Health Organization Collaborating Centre for Research and Training in Suicide Prevention; School of Applied Psychology, Griffith University, Mt Gravatt Campus, Brisbane, QLD 4122 Australia; 2 Child and Youth Mental Health Service, Queensland Children’s Hospital, Brisbane, Australia

**Keywords:** Lived-experience, Young people, Intervention, Suicide prevention, Phenomenology, Mental health

## Abstract

**Background:**

Suicide in young people is a leading cause of death. Interventions that are reflexive, tailored, and developed in concert with this at-risk population are needed. This study aimed to integrate lived-experience into the design of a suicide prevention intervention delivered by phone to young people post-discharge from an emergency department (ED) for suicide risk or self-harm.

**Methods:**

Qualitative study was conducted at the Queensland Children’s Hospital, Brisbane Australia. Four focus groups with young people with lived-experience, parents or carers and ED mental health clinicals were conducted. In total 5 young people with lived-experience of suicidality (17–21 years, *M*_*age*_ = 19.20), 3 parents and carers with a lived-experience of caring for a young person with mental illness, and 10 ED mental health clinicians participated in focus groups. The *first phase* of qualitative analysis involved a phenomenological analysis and *second phase* included a deductive content analysis. The paper is following the Consolidated Criteria for Reporting Qualitative Research.

**Results:**

First phase, a phenomenological analysis identified three foundational themes to structure future follow-up phone interventions: a person-centred focus, the phone-call dynamic, and the phone-call purpose. Second phase, a deductive content analysis found that participants preferred an intervention that was structured, consistent, and finite. Moreover, an intervention that was authentic, able to facilitate and empower growing independence, and achievable of young people after an ED presentation was desired.

**Conclusions:**

Participants expressed their desire for a responsive, structured, and clearly focused phone call that would recognise the young person and parent/carer’s needs while providing tailored support to ease transition from the ED to available community and family led care.

## Introduction

Suicide remains a leading cause of death in adolescents and young adults with some studies showing increasing trends of deliberate self-harm in young people [1–3]. This calls for a critical look into existing treatment practices to develop new interventions.

Transfer of care from emergency settings to outpatient treatment is a key area of patient safety-risk for individuals at risk of suicide. The risk is that Eds function as the primary or sole point of contact within the healthcare system for patients and their families, with young people or families not attending outpatient treatment outside of a suicidal crisis [4]. Follow-up care after an ED presentation is an important intermediary step in care between ED discharge and outpatient mental health services. Indeed, previous studies have shown assertive telephone follow-up of patients at risk of suicide discharged from Eds may reduce the risk of future suicidal behaviour and reduces the risk that patients do not attend outpatient treatment [5–8]. However, most of these interventions have been tested in adult populations [6, 7].

Increasingly, research funding bodies and advocacy organisations require suicide prevention interventions draw upon the expertise of those with lived-experience. Interventions incorporating lived-experience of young people are rare [9, 10]. Lived-experience refers to people with personal experience of suicidal thoughts, surviving a suicide attempt, having cared for someone through a suicidal crisis, or been bereaved by suicide [11]. Interventions integrating this experience are suggested to increase an interventions’ relevance and effectiveness [12, 13].

Our recent scoping review identified only 11 studies reporting the development of suicide prevention interventions incorporating lived-experience [10]. Most of the studies employed focus groups and participatory workshops to generate in-depth understanding of the concerns and preferences of people who had experienced suicidal behaviour themselves or in their families, frontline mental health clinicians, or researchers in this sphere [10]. However, only a few papers discussed the methods utilised in intervention development and there was limited discussion of the translation from qualitative themes to the intervention activities.

The current study aimed to integrate lived-experience into development and refinement of a suicide prevention intervention for young people (aged 17–25 years) delivered as a follow-up phone-call post-discharge from an ED. Young people with lived-experience of mental illness, parents/carers with lived-experience of caring of a young person with mental illness, and frontline mental health clinicians working in the ED were recruited to offer their perspectives.

## Methods

A qualitative design, implementing focus groups was employed for this study. Focus groups on healthcare research have several advantages relevant to the current research study. The group setting encourages active participation from individuals who may be reluctant to be interviewed or voice their opinion on their own, facilitates the discussion of taboo topics, and allows participants to mutually support one another in expressing views that may deviate from that assumed of the research team [14]. The paper is following the Consolidated Criteria for Reporting Qualitative Research [15]; Appendix 1).

### Sample size justification

Phenomenological qualitative studies require small samples of up to 10 cases for a thematic analysis [16]. Furthermore, research suggests that 80% of all themes are identified within two to three focus groups, and 90% are identified within three to six focus groups. Therefore, we aimed for 10 participants per participant group across three to six focus groups [17].

### Participant selection

Three groups of participants with lived-experience was recruited: young people, carers, and mental health clinicians working in ED. The study was limited to the consumer advisory groups (consumers/community members involved in assisting the hospital to plan, design and deliver better health services for children and young people) and Acute Response Team (a 24 h ED-located team providing mental health triage and assessment to children and young people) and advertised at regular meetings. Recruitment emails were sent out by the convenors of the relevant groups (the Children’s Health Queensland CYMHS Beautiful Minds and the CYMHS Parent/Carer Advisory Group). Mental health clinicians were invited to participate on a voluntary basis, it was emphasised that there were no detrimental effects for clinicians who declined to participate and that transcripts would be de-identified (given that two members of the research team also worked in the CYMHS). Recruitment was limited to young people (aged 17–25 years) with lived-experience of discharge from the Queensland Children’s Hospital ED after a suicide attempt or self-harm incident, lived-experience caring for a young person who had been discharged from the ED following a suicide attempt or self-harm incident, or a mental health clinician working in the ED.

### Data collection

Focus groups were conducted between April and May 2019 at the Queensland Children’s Hospital campus. Written consent was obtained prior to focus groups beginning. For participants under the age of 18 years, written consent was also obtained from a parent. During and immediately after the focus group, a clinician was available for support if a participant became distressed; no such events occurred during the study. All focus groups were conducted in conference rooms with refreshments provided. Focus groups were conducted separately for each type of stakeholder group with two focus groups being held for mental health clinicians, one for young people, and one for carers and parents. Participants completed a demographic questionnaire asking their age and gender. Focus groups were conducted separately for each type of participant to capitalise on individuals’ shared experiences. The homogenous group setting encourages active participation from individuals who may be reluctant to be interviewed or voice their opinion on their own, facilitates the discussion of taboo topics, and allows participants to mutually support one another in expressing views that may deviate from that assumed of the research team [18].

The focus groups were conducted by a female clinical psychologist with more than 15 years of experience in child and youth mental health and suicidality, who was assisted by a male PhD candidate in psychology. The first (experienced in conducting focus groups) led the focus group discussion following the interview booklet and kept participants on track. The second researcher assisted by observing, taking notes, and monitoring the audio recording device. Neither had previously met any of the participants and spent the start of each focus group establishing a rapport through general conversation and explaining the study’s purpose and personal biases. The research team members who were involved in recruiting participants welcomed the participants to focus groups but left prior to focus groups beginning.

Young people and carers were reimbursed for their time commitment (of up to 3 h) with an AUD$110 gift card. Mental health clinicians were offered the convenience of participating in the focus group during work time and at their workplace.

All focus groups lasted approximately 90 min and were audio recorded. Recordings were transcribed by Pacific Transcriptions with all participants de-identified. All participants were offered the opportunity to review the de-identified transcripts.

### Ethics

All procedures were approved by the Children’s Health Queensland (HREC/18/QCHQ/44615) and Griffith University Human Research Ethics Committees (GU HREC 2018/990). By our ethical clearance we were limited to recruit participants of the Children’s Health Queensland CYMHS Beautiful Minds and CYMHS Parent/Carer Advisory Groups, who were supposed to remain anonymous.

### Interview guide

A semi-structured guide was developed after review of similar projects available in the literature and through clinical experience (Appendix 2). Briefly, however, the questions primarily focused on three distinct models of follow-up interventions (an existing unstructured model; assertive [7]; caring contacts [19]) post-discharge from the ED. A description of each model is provided in Appendix 3. Questions were asked about participants’ perception of a follow-up phone call service, what was useful or needed to be changed from each of the three interventions, helpful messages, and finally a section for open comments. This final section of the focus group allowed participants to suggest alternative solutions to follow-up care.

### Participants’ demographics

Clinicians consisted clinical nurses (*n* = 5), social workers (*n* = 3), or psychologists (*n* = 2) and had between 6 and 20 years of experience in mental health (*M*_*exp*_ = 11.75, *SD*_*exp*_ = 5.17). Ten clinicians (six female) participated across two focus groups (aged 27–47, M = 38.40, SD = 7.07). Five young people (3 male) participated (aged 17–21, M = 19.20, SD = 1.47) and three female parents/carers participated (57–65, M = 58.33, SD = 4.99) in separate focus groups.

### Data analysis

The beginning of the qualitative analysis was creating a foundation for an intervention to be built upon. While funding, effective counselling methods, service access, and target populations may change over time, a solid foundation that reflects the lived-experience can provide the platform for a range of intervention iterations. To achieve this, the *first phase* of qualitative analysis involved a phenomenological analysis using the seven steps described by Colaizzi [20] using Nvivo (version 12). This method has previously been used to understand and represent the lived-experience of people across a range of experiences and was a logical method given the aim was to understand participants’ lived-experience of suicidal behaviour [21–24]. The following seven-steps were conducted:

1. Familiarised and immersed in the transcripts (conducted by all authors);
2. Identified all significant statements relevant to the phenomenon of receiving phone calls after ED discharge (DW, MP);
3. Identified meanings from the relevant significant statements (DW, MP);
4. Clustered meanings into similar themes into a computer word processor (DW, MP);
5. Produced an exhaustive description of phenomenon with all themes (DW);
6. Produced condensed description of primary aspects of the phenomenon (DW); and
7. Returned the structure statement to all participants to ensure it captured their experience (DW).

The final step of the Colaizzi [20] method has not been endorsed by some due to the theoretical and practical concerns (e.g., [25]), however, this step was deemed crucial from the lived-experience involvement perspective and was therefore included in the current study. Inclusion of this final step meant that we were able to ensure those with lived-experience were included as far as practical throughout the study and write-up. A small number of young people (*n* = 2 who had completed the focus groups and *n* = 2 who had not), and parents/carers (*n* = 3) were consulted to discuss their satisfaction with the validity of the results. All of those consulted were satisfied with the accuracy of the interpretation of their experience.

Acknowledging that phase one provides only a foundation for the intervention and does not structure physical ‘content’, *phase two* aimed to develop this. While guided by clinical experience (SB; MP; JH), phase two involved a secondary qualitative analysis to help develop content for the intervention and analyse participants’ practical suggestions. As such, a deductive content analysis was conducted based loosely around the semi-structured focus group questions. For example, focus group questions broadly assessed what participants thought was helpful or what should be changed about existing post-discharge follow-up interventions; utility of the existing (phone call) intervention; preferences for intervention target and content; timing of intervention; and finally, messages that may be helpful to others in this situation (i.e., post-discharge). With this overarching framework in place, a deductive content analysis (as per [26] was applied to participants’ responses in relation to questions that were asked throughout the focus groups (e.g., “What do you think could be of help in this model?” and “If you could change anything about this model what would it be?”. This method is appropriate to help generate further structure and content to the intervention that was responsive to the participants with lived-experience [26]. All researchers separately familiarised themselves with the data, identified significant statements, and collated into overarching potential themes in relation to the focus group questions. All researchers then met to discuss preliminary themes and two researchers (DW, MP) progressed this separately into final themes that would inform intervention content.

## Results

### Phase one

A summary of the results of steps two to four from Colaizzi [20] phenomenological method is provided in Table 1. Three foundational themes were identified that best represented participants’ lived-experience including *a person-centred focus*, *phone call dynamic*, and *phone call purpose*. A description of each theme is provided below (i.e., Step 5) with example statements presented in Table 2. A condensed overarching statement capturing the experience of phone calls to participants (i.e., step six) is provided at the conclusion of phase one results. Initially, themes were explored for each participant group separately, however, due to the small sample and the similarity of responses across groups, a comparison between participants was not included. Nonetheless, responses from each group are presented separately in Table 1 while summaries of all groups have been synthesised below.

**Table 1:** Steps two to four by Colaizzi’s [19] seven step phenomenological qualitative approach to develop intervention foundational themes

Participant	Step 2: significant statements	Step 3: formulated meanings	Step 4: themes
Clinician	(1) “It's really hard to kind of provide a generic kind of template around that, because you do it—like you've generally reviewed the notes and you're tailoring the conversation to what the assessment was, so it's hard to say, oh yes, I'd do this and then I'd do that.”	(1) Follow-up interventions need to be responsive/tailored to the individual, as opposed to a one-size-fits-all approach	(1) Person-focused: The intervention needs a strong person-centred focus
	(2) “It's a bit kind of like, yeah, just building a banter with them and a therapeutic rapport and then albeit briefly, then being able to dive into those harder questions.”	(2) A genuine, empathic, and rapport rapport-building intervention must be prioritised over a 'tick the box' approach	(2) Phone call dynamics: Ensuring a genuine and empathic call is of primary importance
	(3) “I feel it would be helpful, but there would need to be a purpose of the call, like rather than, oh how are you feeling today, yeah, still suicidal, oh that's shit. Like more of a have you called your GP like you said you would, have you followed like we tell the parents to remove access, have you done this with the parents, like more of a call to follow through and have you put into place what we discussed.”	(3) The intervention purpose must be clear and aim to meet the expectations and wants/needs of the individual	(3) Phone call purpose: The purpose of the call to each person must be clear
Young person	(1) “I think it depends on the circumstances."		
	(2) “The person is still human. Just because they have a mental illness doesn’t mean they're any less mentally capable than anyone else. In my experience, when I've ever talked to someone over a phone I feel like they're reading from a script and I'm talking to someone that's robotic, and I totally lose all connection. It's, like, okay, let's wrap this up, I've got things to do.”		
	(3) “I think psychoeducation is a big part of it. I know for my parents they didn’t really understand what I was going through. To them when I was really young it was like, this is just attention or this is just—why didn’t you talk to us about this sooner? Just not really getting it. If someone had taken the time to sit down with them and say, do you actually get how we got to this point—because they didn’t see the signs leading up to the attempt so they were just like, oh, this just happened. If someone had sat down with them and said, did you notice this, this and this? Because that was what that was, and this is what is happening with your child. They might have understood a bit better and been more caring about it.”		
Parent/carer	(1) “Generally, yes, but that should be stated at—while you're in the ED. I've asked my daughters about this and they said that they would like to know in advance to have the right to say yes, they want someone or no, they don't.”		
	(2) “They don't want that specific have you decided to take anymore? Have you—not that they would ask that but they don't want that specific to—they feel like they're more confronted if they have to answer those questions. They just want to round it out, which my son still gets when he goes to his clinicians…”		
	(3) “I agree. It's terribly important to follow-up but as kids get older you really have to take in their thinking about it all. As a parent when we needed to follow-up with a youngster, like 12, that was my lifeline, having someone call me. It was very important to me.”

**Table 2:** Foundational themes with example statements from each participant group

Clinician	Young person	Parent/carer
Person-centred focus		
*“I think you kind of gauge that case by case when you go down and you actually talk to parents, like…”*	*“I think it depends on the circumstances. Going back a few steps, if you've identified with the young person that there's no risky stuff going on that their family is putting them at risk or is damaging their mental health then I think that's a good idea. But I think also realising that's a different kind of clinical discussion you're having than the discussion with the young person themselves”*	*“Generally, yes, but that should be stated at—while you're in the ED. I've asked my daughters about this and they said that they would like to know in advance to have the right to say yes, they want someone or no, they don't.”*
*“It's really hard to kind of provide a generic kind of template around that, because you do it—like you've generally reviewed the notes and you're tailoring the conversation to what the assessment was, so it's hard to say, oh yes, I'd do this and then I'd do that.”*	*“I would say definitely contacting the young person first is going to be important, because imagine if you're the young person and your family is contacted before you about this, and you're told by your mother or your father, they just called me about this. You weren’t told about this. You weren’t asked any questions directly.”*	*“It would be helpful also if they could specify some sort of timeframe of when they're going to…”*
Phone call dynamics		
*“It's a bit kind of like, yeah, just building a banter with them and a therapeutic rapport and then albeit briefly, then being able to dive into those harder questions.”*	*“Yeah. When they're, like, so when I saw you we talked about this, and I was, like, you remember who I am. That's nice. I'm a human. As opposed to, blah, blah, blah.”*	*“Often in a crisis, you don't think of everything that needs to be said and that there is follow-up emergency stuff that really unanswered questions that needs to be addressed.”*
*“I guess a conversation directly around risk or if they were in hospital previously [around] risk taking their life and how, where are they feeling now … yeah.”*	*“I feel like it's got to do as well with connection. For me, if don’t have a connection with someone, I'm not going to tell them anything. But if I can at least trust them a little bit and build a little bit of rapport, I'm going to be a lot more open. Maybe just try and build that rapport, talk about something other than the visits. Like, have you seen a movie today? What have you, I don’t know, eaten?*	*“…should have a checklist that they go through themselves and they can point out on each—the person who rings up can also have that same checklist and they can refer different questions to A, B, whatever and say this is happening. What do you think I should do? Should I go and see someone? Should I just leave it and wait and see?”*
Phone call purpose		
*“I feel it would be helpful, but there would need to be a purpose of the call, like rather than, oh how are you feeling today, yeah, still suicidal, oh that's shit. Like more of a have you called your GP like you said you would, have you followed like we tell the parents to remove access, have you done this with the parents, like more of a call to follow through and have you put into place what we discussed.”*	*“I think psychoeducation is a big part of it. I know for my parents they didn’t really understand what I was going through… They might have understood a bit better and been more caring about it.”*	*“Often in a crisis, you don't think of everything that needs to be said and that there is follow-up emergency stuff that really unanswered questions that needs to be addressed.”*
*“We're just trying to make sure that they know how to access support, counselling if they need it, so what our number is, if this happens again how to proactively get help when they need it.”*	*“How they're going. Check in on them.”*	

### Person-centred focus

The most evident theme that was identified from the transcripts was the notion of ensuring a *person-centred focus*. All participants were highly in favour of ensuring that regardless of the intervention design, the approach should be person-centred and participatory in nature. Participants raised the idea of being heard, understood, and being active in the intervention process. Common words that were raised indicating the person-centred focus included ‘wants’, ‘preferences’, ‘tailored’ and this focus appeared across all participant groups. For example, clinicians often expressed the notion that a follow-up call would depend on clinical experience and the presenting young person. They expressed a preference not to have a blanket approach, but to use clinical judgement around unique presentations and needs of the client and their situation. Young people expressed their thoughts in terms of being an active participant in the process. They spoke of their preference to have the service tailored to them and their personal situation, inferring the notion that they hoped to take some control over the situation. Carers demonstrated their desire for a person-centred focus by requesting information they could use to gain some control over the situation (e.g., when contact would be made; Table 2).

### Phone-call dynamics

A second, related theme was the *phone-call* or follow-up service *dynamics*. This theme was clear across all participant groups, with key words expressing the need for ‘rapport’, ‘relationship building’, and developing a ‘connection’ with participants. Clinicians’ focus in this area was around trying to break the ice and form a connection to facilitate the clinical discussion and more difficult questions that would present (e.g., thoughts of suicide). Perhaps complimentary to the views of clinicians, consumers expressed their need for ‘empathy’, speaking to a ‘genuine’ clinician, and having open communication. With this approach, the young people suggested they would feel validated and may be more likely to engage (Table 2). Carers similarly expressed a need for support and connection but extended this into a more practical form with common statements around being ‘supported’ and having something ‘concrete’ they could refer to in the future.

### Phone-call purpose

The final foundational theme evident across all groups was ensuring a very clear understanding around the *phone call purpose* and ensuring that all parties receive the appropriate support. For example, it became evident for clinicians, that resourcing was stretched. As such, having a clear layout of what the call must include may enhance the viability, and efficacy of the call (Table 2). Young people expressed similar preferences, identifying that they would like the phone call to operate as a ‘check-in’ service, or one that could provide support and guidance at this time. Moreover, the young people identified the importance of passing on information to the family members (i.e., psychoeducation) which would in turn better facilitate the family to understand and identify warning signs. In line with this perspective, the carers also expressed their desire to receive information and support from the call. One parent framed the follow-up phone call as a ‘lifeline’ at this extremely challenging time where most parents see themselves as ill-equipped to effectively manage the situation. Moreover, a common statement was not having the presence of mind when the young person is being discharged to ask all the questions that will need answering over the following hours. It was this desire for information that dominated the discussion for the parents.

### Statement of phenomenon (step 6)

Phone calls post-discharge from the ED need to be responsive to the person and situation. Each phone call must be structured, with a clear purpose recognising the individual’s needs and ensuring that an empathic understanding of the young person is created to facilitate open and honest communication. The service must also provide tailored support and guidance to ensure a smooth transition of care from the ED to available community and family led care.

#### Phase two

Combining the focus group questions that asked participants: (a) what they liked about the three presented models; and (b) what they would change about the models, the overarching themes of ‘what works’ and ‘what does not work’, respectively, were identified (Table 3). Participants’ preferences for a follow-up intervention centred on it being structured, consistent, and finite. In combination with the foundational concepts, this structure could be adapted with clear communication at discharge about what is involved in the intervention. Participants broadly agreed that an intervention must be authentic and facilitate and empower growing independence. Moreover, the intervention must have a clear and achievable aim.

**Table 3:** Deductive analysis of transcripts for intervention content

Theme	Description	Key quotes
What works		
Structure	Participants discussed importance of a comprehensive, structured, and reliable phone call that provided assessment (where possible) of key outcomes and risk factors (e.g., mood) as well as supporting the young person and the carers with problem-solving techniques or advice and being able to facilitate appointments with community services	*“But I also like how it goes over the mood stuff and suicide risk assessment with the safety planning and then…” [Clinician—Assertive Model]*
		*“I feel it's nice to go, hey, have you made it to your appointment? Okay, let's troubleshoot what happened, what stopped you from going. Let's sort that out. But again it does have that thing about, what if there's an insurmountable barrier stopping you from taking care of yourself. But I like that it's, we're going to—not just, have you been? What's happening there? How do we sort it out so that you can go?” [Young person—Assertive Model]*
		*“Or, you haven’t used your safety plan? Is that because you're fine or because it's not working for you? Do we need to change something about it?” [Young person—Assertive Model]*
		*“structure around questions or what we're checking on, like…” [Clinician—Assertive Model]*
Consistency	Participants and clinicians expressed desire for an intervention that was consistent, but able to be adapted as needed (i.e., tailored to the individual), and one that could be counted on was preferred (e.g., consistent clinician where possible)	*“at the moment I think different clinicians have very different processes [unclear] so it provides consistency…” [Clinician—Assertive Model]*
		*“that reassurance that things are going to be okay and there is somewhere to go and someone to call…when things get tough again. So I think it's helpful in that sense.” [Clinician—Assertive Model]*
		*“I think clinicians want it, they want that level to be able to do appropriate follow-up like that, we want that sort of stock standard response, we want to be able to say yes, we're going to provide this.” [Clinician—Assertive Model]*
		*“This takes all that sort of unknown away and it's this [unclear] organisation that's there if you need us.” [Clinician—Caring Contacts]*
		*“The contact. The actual contact opens conversation” [Young person—Assertive Model]*
Contained/finite	Satisfaction was expressed with models that were clear and contained (as opposed to ongoing with fuzzy boundaries). Comments showed preferences for a model that would have an endpoint and support the young person to progress back into community and family-led care	*“This is good, it's contained, it's got clear guidelines [with the] 72 h. It's one phone call and these are the points that we're going to hit.” [Clinician—Assertive Model]*
		*“like it's got a finite amount of calls and it's spaced out enough that it's not a stalker-ish kind of setup” [Clinician—Assertive Model]*
		*“in terms of a system as well it's less work for us, because I think if we have all these [supportful] letters and postcards and text messages already set out, so then you can send it out …” [Clinician—Caring Contacts]*
		*“I think in terms of workload for us that is by far the easiest option.” [Clinician—Caring Contacts]*
What does not work		
Practicality	While the structure and content of the assertive model was appealing, and the empathic nature of caring contacts reassuring, there was doubt expressed around whether each model would be practical to implement. Clinicians’ comments centred on the extensive requirements and the concern around whether accurate assessment of outcomes could be made. Perceptions of the caring contacts model were positive, but agreement on the model losing authenticity over time and potentially confusing the follow-up services’ purpose (i.e., a key foundational requirement; see phase 1):	*“In an acute team that's 24/7, I'd feel like we're still getting lost.” [Clinician—Assertive Model]*
		*“First of all, what if the young person has already used their 10 sessions for the year and it's September? Are you going to give them a weekly phone call until January? Because that seems like a really resource-heavy way of doing things, and I feel that's not going to happen in Q Health.” [Young person—Assertive Model]*
		*“on paper this is a really good idea but it has the capability to be really quickly, I'm going to say the word, delegitimised.” [Young person—Caring Contacts]*
		*“we don't want to be the ones that build the ongoing therapeutic relationship.” [Young person—Caring Contacts]*
Disempowering	Participants expressed concern around possibility of disempowering the young person and their family, particularly in response to the assertive model. Clinicians cautioned that there must be a clear separation from their services into the hands of the parents and guardians or community services (may be more difficult with more intensive interventions). Young people perceived structured, and highly detailed interventions as intense (may run the risk of taking control and autonomy away from the individual). Sentiment also verbalised by the carers and returns to the notion of a person-centred foundation	*“… elements of it, but the onus has got to go back to the family too make their own follow-up.” [Clinician—Assertive]*
		*“Support them as much as you possibly can, I agree with you 100 per cent, but the onus has got to go back to the family at some point.” [Clinician—Assertive]*
		*“I think with this it’s 0 to 100 real quick. There’s no middle ground, and I think that is potentially a mistake.” [Young person—Assertive]*
		*“She has to be involved. You can’t do a safety—we can’t give her a safety plan.” [Parent/Guardian—Assertive]*
Call purpose	Clinicians expressed need for clear boundaries (assertive and caring contacts models) and the notion that calls should facilitate connection with community services (rather than continuing contact with the emergency response unit)	*“Well state your purpose.” [Clinician—Assertive Model]*
		*“…what's the purpose of the call, yeah, are they always suicidal?” [Clinician—Assertive Model]*
		*“you're asking people to come in and build a relationship with that clinician rather than the community clinician.” [Clinician—Caring Contacts]*
		*“… we're a crisis team, it's just … people who are in there, then once we see them we need to move them on to someone else.” [Clinician—Caring Contacts]*
Helpful messages	Two primary themes emerged from suggestions which can be used as a template to generate messages. ‘Validating the person and their experience’ as well as ‘normalising the experience’ were broad focus areas along with ensuring the messages were ‘person-focused’. General suggestions on the type of message that could be sent (e.g., providing advice on who to speak with, what to do in risky situations, or how their discharge/safety plan is going)	*“You did really good the other night chatting to us or calming down” [Clinician]*
		*“I can see a really resilient young person and you were really motivated to engage in therapy, that should be applauded, if you don't have hope for yourself, I have hope for you.” [Clinician]*
		*“I'll hold the hope for you.” [Clinician]*
		*“We know you've been discharged and you've been travelling quite well. If you have any concerns feel free to give us a call.” [Clinician]*
		*“A journey and having bumpy parts in the road or whatever” [Clinician]*
		*“I'm sorry that this sucks”[Young Person]*
		*“Out of suffering have emerged the strongest souls. The most massive characters are seared with scars” (Poetry quotation)[Young Person]*

Helpful and supportive messages developed by participants were focused around two primary themes, ‘validating the person and their experience’ and ‘normalising the experience’ (Table 3). Participants also expressed suggestions for the types of message to be sent (e.g., providing advice on who to speak with, what to do in risky situations, or how their discharge/safety plan is going). As such, the broad focus areas raised can be used as a template for future message creation.

## Discussion

Brief, suicide prevention interventions, and those provided in clinical settings (e.g., an ED) have shown positive effects on repeated self-harm and suicide attempts (e.g., [9, 27]). Moreover, the importance of incorporating lived-experience into intervention development is clear ([12, 13, 28]). To incorporate lived-experience into a suicide prevention intervention for young people [29], this study aimed to explore young persons’, parents/carers’, and mental health clinicians’ lived-experience to develop and refine a brief intervention delivered as a follow-up phone-call post ED discharge. A person-based approach to intervention development (e.g. [30]), was taken to increase the likelihood of the resultant intervention being relevant and persuasive [10, 30]. The current study represents *planning* (focus groups with stakeholders) and *design* (intervention components from transcripts) stages of intervention development from the O’Cathain, Croot [30] person-based approach.

Across two phases of qualitative analysis, intervention foundations and content themes were derived from focus groups with young people, parents/carers, and mental health clinicians with relevant lived-experience. A phenomenological approach (inductive) was utilised to derive main themes for intervention foundations and a deductive content approach was used to derive intervention content related themes structured around interview questions. Across all focus groups, three foundational themes were identified: the need for a patient-centred focus, a strong desire for support and connection, and a clear understanding and communication of the purpose of the follow-up service. Phone calls post-discharge from the mental health service need to be responsive to the person and situation (i.e., person-focused). Rather than a ‘check-the-box’ procedure, the call should be responsive to the person and their needs and be organised collaboratively. Second, the intervention must prioritise building rapport and developing a working relationship as this will increase the likelihood of connecting with the person. Finally, the intervention must have a clear purpose that is communicated with the user, for example, a ‘check-in’ service where appropriate supports (e.g., psychoeducation, community-care referrals) are provided. With these pillars as a foundation, content can be adapted and structured to suit the individuals’ needs.

Building upon the foundational themes, a secondary round of deductive analysis of transcripts identified components that users thought would work and those that would not in the intervention. All participants expressed positivity for a structured (i.e., relevant assessments, education), consistent (i.e., reliable contact schedule, consistent clinician), and contained/finite intervention (i.e., manageable, clear end point). In addition, there was concern around the practicality (i.e., feasible, achievable), the risk of disempowering (i.e., taking control and choice away from users), and losing focus of the call purpose (i.e., clear boundaries, referrals). Inclusive of these content themes, an intervention outline has been proposed, which aligns with the results of the qualitative analysis.

Previous work integrating lived-experience into suicide prevention interventions have provided limited detail around translating findings into the physical intervention [10]. Addressing this limitation, the current paper provides greater detail around this process while ensuring recommendations by key bodies in the suicide prevention sphere are achieved (e.g., person-based, lived-experience; [29]. Integrating the lived-experience of young people, parents/carers, and mental health clinicians increases the likelihood of the intervention being relevant, persuasive, and engaging [30]. Moreover, the identification of three foundational themes may be beneficial to future iterations of the intervention where funding and service delivery may change. The identified themes can be implemented as a foundation for a range of interventions regardless of the format (e.g., phone, web, or app-based). To continue the development and refinement of the intervention, a pilot study will be conducted to assess the feasibility and acceptability of the intervention.

### Intervention development

Integrating the current findings into a newly designed post ED-discharge phone-call intervention is currently underway. Overall, the involvement of lived-experience guided us towards incorporating aspects of the existing model and the assertive model into the new intervention. While the caring contacts model was seen to have promise, it was viewed very differently by the young people as opposed to the mental health clinicians. Young people saw ‘caring contacts’ messages as only worthwhile when the messages were meaningfully constructed for a particular individual and truly empathic, whereas the mental health clinicians were eager to fully automate such messages. Given the diverging views of the caring contacts model, we saw its inclusion in our new intervention as a risky prospect.

Currently, the protocol of the new intervention comprises a series of phone calls to a family (comprising the recently discharged patient and their parent/carer) until the patient attends their first outpatient appointment after an ED presentation. We have developed a template including tasks to be completed by the clinician in the calls (clarifying the purpose of the call), but with the overarching themes that the calls be person-centred and build a connection. A large part of the new intervention is greater transparency with families, such that they receive a greater explanation of the role of phone calls while still in the ED and have an opportunity to have it tailored to their individual circumstances at the time of set-up (e.g., families negotiating with the clinician what day and general time a call will be made, the young person being able to say if they want to be a part of the call or not, etc.). As such, when aligned with the foundational themes to fluidly respond to each young person’s and their family’s needs, the intervention can still maintain important structures to achieve the purpose of the follow-up contact.

## Methodological considerations and future directions

The current study has a relatively small sample size, which might be limiting the depth and breadth of reflections; however, its sample size is comparable to previous studies [10, 31, 32]. There may be commonalities in opinions of the mental health clinicians (as they work in the same service) and the female parents and carers of young people (no fathers or male guardians participated), perhaps limiting generalisability to other hospitals and families. However, the study’s aim was to develop and refine an intervention contextualised for this Queensland Children’s Hospital; thus, sampling within the hospital’s clinicians and carer groups was considered as appropriate. Nevertheless, our sample size was impacted by our recruitment strategy which was limited to the Children’s Health Queensland CYMHS Beautiful Minds and CYMHS Parent/Carer Advisory Groups. It is important to note that we did not utilise strict co-design approach and used focus groups, nevertheless, we did include the members of the consumer groups involved throughout the study and they did give feedback to the final themes and to the intervention development.

While every attempt was made to ensure participants were free to express their own views (e.g., by holding separate focus groups for each type of stakeholder), less confident or less outspoken individuals may not have been as forthcoming in expressing their true opinions. Moreover, the sample was weighted towards clinicians which may unduly bias our results however, we did analyse our results on group level to avoid overrepresentation of clinicians’ views. Future work may explore individual interviews to supplement the focus groups, and across multiple settings to ensure a larger sample.

Furthermore, while there was provision for participants to freely explore their own views towards the end of the focus groups, the semi-structured approach may have indirectly influenced the discussion. This could be avoided by encouraging free exploration in the beginning of the focus groups. However, free exploration may have been more temporally demanding on participants, and less economical in terms of information obtained, compared to the methodology employed. The focus group topic guide was weighted towards a follow-up telephone call intervention based on the background literature review, which was considered suitable for the metropolitan ED setting considering the resources, rather than other types of interventions. Further research is needed to test the feasibility of the newly designed intervention, attending to both its acceptability to young people, their families, and clinicians. Moreover, intervention effectiveness (clinical and economic) will need to be evaluated in large scale studies.

## Conclusion

This qualitative study employed an empirically supported method [10, 30] to understand the lived-experience of a post ED discharge follow-up suicide prevention phone call with young people, parents and guardians of young people, and front-line mental health clinicians. This process helped to develop a foundation for an improved and tailored follow-up phone intervention. Key facets of the new intervention will include a responsive, structured, and clearly focused phone call.

## Data Availability

De-identified participant transcripts can be provided upon request for research purposes with further requests from the relevant HRECs.

## Consolidated criteria for reporting qualitative studies (COREQ): 32-item checklist

Developed from:

Tong A, Sainsbury P, Craig J. Consolidated criteria for reporting qualitative research (COREQ): a 32-item checklist for interviews and focus groups. *International Journal for Quality in Health Care*. 2007. Volume 19, Number 6: pp. 349 – 357.

YOU MUST PROVIDE A RESPONSE FOR ALL ITEMS. ENTER N/A IF NOT APPLICABLE

**Table:**

No. item	Guide questions/description	Reported on page #
Domain 1: research team and reflexivity		
Personal characteristics		
1. Interviewer/facilitator	Which author/s conducted the interview or focus group?	8
2. Credentials	What were the researcher’s credentials? e.g. PhD, MD	8
3. Occupation	What was their occupation at the time of the study?	8
4. Gender	Was the researcher male or female?	8
5. Experience and training	What experience or training did the researcher have?	8
Relationship with participants		
6. Relationship established	Was a relationship established prior to study commencement?	8
7. Participant knowledge of the interviewer	What did the participants know about the researcher? e.g. personal goals, reasons for doing the research	8
8. Interviewer characteristics	What characteristics were reported about the inter viewer/facilitator? e.g. Bias, assumptions, reasons and interests in the research topic	8
Domain 2: study design		
Theoretical framework		
9. Methodological orientation and Theory	What methodological orientation was stated to underpin the study? e.g. grounded theory, discourse analysis, ethnography, phenomenology, content analysis	10
Participant selection		
10. Sampling	How were participants selected? e.g. purposive, convenience, consecutive, snowball	7
11. Method of approach	How were participants approached? e.g. face-to-face, telephone, mail, email	7
12. Sample size	How many participants were in the study?	9
13. Non-participation	How many people refused to participate or dropped out? Reasons?	n/a
Setting		
14. Setting of data collection	Where was the data collected? e.g. home, clinic, workplace	8
15. Presence of non-participants	Was anyone else present besides the participants and researchers?	8
16. Description of sample	What are the important characteristics of the sample? e.g. demographic data, date	9
Data collection		
17. Interview guide	Were questions, prompts, guides provided by the authors? Was it pilot tested?	9
18. Repeat interviews	Were repeat inter views carried out? If yes, how many?	8
19. Audio/visual recording	Did the research use audio or visual recording to collect the data?	8
20. Field notes	Were field notes made during and/or after the inter view or focus group?	8
21. Duration	What was the duration of the inter views or focus group?	9
22. Data saturation	Was data saturation discussed?	n/a
23. Transcripts returned	Were transcripts returned to participants for comment and/or correction?	9
Domain 3: analysis and findings		
Data analysis		
24. Number of data coders	How many data coders coded the data?	10
25. Description of the coding tree	Did authors provide a description of the coding tree?	10
26. Derivation of themes	Were themes identified in advance or derived from the data?	10
27. Software	What software, if applicable, was used to manage the data?	9
28. Participant checking	Did participants provide feedback on the findings?	10–11
Reporting		
29. Quotations presented	Were participant quotations presented to illustrate the themes/findings? Was each quotation identified? e.g. participant number	Tables 2–4
30. Data and findings consistent	Was there consistency between the data presented and the findings?	10–11
31. Clarity of major themes	Were major themes clearly presented in the findings?	12–15
32. Clarity of minor themes	Is there a description of diverse cases or discussion of minor themes?	14–15

Once you have completed this checklist, please save a copy and upload it as part of your submission. When requested to do so as part of the upload process, please select the file type: *Checklist*. You will NOT be able to proceed with submission unless the checklist has been uploaded. Please DO NOT include this checklist as part of the main manuscript document. It must be uploaded as a separate file.

## List of prompts and questions for the focus groups

1. Would it be helpful for a mental health clinician to call a family after being discharged home from the emergency department for suicide risk?
  - How would it be helpful?
  - What could the clinician ask the parent/carer or say to the parent/carer to make it helpful?
  - What could the clinician say to the young person to make it helpful?
2. Currently, the Child and Youth Mental Health Service (CYMHS) Acute Response Team provides limited telephone follow-up to patients at risk of suicide discharged from the emergency department for up to one week following discharge. Families typically receive maximum 4 telephone calls with no set guidelines around who specifically is called or what needs to be discussed in the call. Some clinicians call the parent/carer only, some clinicians call the young person only, some clinicians call and speak to both the parent/carer and young person, and some families are not scheduled for calls. Calls are often not made by the same clinician who met the family in the emergency department due to shift-work patterns. Calls are typically made in the late afternoon and early evening. This *current model* of telephone follow-up has developed over time based on available resources and clinician preference:
  - What are your thoughts about this *current model* of telephone follow-up?
  - Remembering that calls are often not made by the same clinician who met the family in the emergency department due to shift-work patterns, what are your thoughts about whether telephone calls should primarily target the parents/carer, the young person, or both?
  - Remembering that calls are typically made in the late afternoon and early evening, what are your thoughts about what time of day is best to make telephone calls? Would your answer differ for parents/carers and the young person?
  - What are your thoughts about young people being routinely asked about thoughts of suicide in a follow-up call? Are there any other questions that might be more comfortable or helpful to a young person? For example, “have you had times where you needed to use your safety plan today?”
  - If you could change anything about this model what would that be?
  - What do you think could be of help in this model?
3. Researchers have developed and implemented what’s called the *assertive telephone follow-up intervention*. This is where families receive 1 telephone call within 72 h of discharge from the emergency department, and then weekly calls until the patient has attended two scheduled outpatient treatment appointments in a row. These researchers suggest that calls include: brief mood check and suicide risk assessment, safety plan review and revision, reiteration of the importance of lethal means restriction, reiteration of the plan for outpatient treatment developed in the emergency department, enhancing treatment motivation through problem solving of any obstacles to treatment, and providing additional referrals as needed:
  - What are your thoughts about this *assertive telephone follow-up intervention*?
  - If you could change anything about this model what would that be?
  - What do you think could be of help in this model?
4. Another group of researchers have developed and implemented what’s called the *caring contacts intervention*. This is where the patient receives caring and hopeful letters, postcards or text messages from the clinician they saw in the emergency department at set intervals (usually no more often than weekly) for a period of time after discharge from the emergency department. An example message is “It has been some time since you were here at the hospital, and we hope things are going well for you. If you wish to drop us a note we would be glad to hear from you”:
  - What are your thoughts about this *caring contacts intervention*?
  - If you could change anything about this model what would that be?
  - What do you think could be of help in this model?
5. Which of the above three models do you believe would be the most helpful and for what reason?
6. Would young people themselves like to be part of an *assertive telephone follow-up intervention* only, to be part of a *caring contacts intervention* only, or to receive both interventions?
7. Thinking about the caring contacts intervention, can you create some caring or hopeful messages that you think a young person would like to receive after being discharged from the Emergency Department?
8. Do you have any final comments about follow-up care after being discharged from the emergency department or about this project?

## Description of models as presented to participants in focus groups

### Current model

Currently, the Child and Youth Mental Health Service (CYMHS) Acute Response Team provides limited telephone follow-up to patients at risk of suicide discharged from the emergency department for up to one week following discharge. Families typically receive maximum 4 telephone calls with no set guidelines around who specifically is called or what needs to be discussed in the call. Some clinicians call the parent/carer only, some clinicians call the young person only, some clinicians call and speak to both the parent/carer and young person, and some families are not scheduled for calls. Calls are often not made by the same clinician who met the family in the emergency department due to shift-work patterns. Calls are typically made in the late afternoon and early evening. This *current model* of telephone follow-up has developed over time based on available resources and clinician preference.

### Assertive

Researchers have developed and implemented what’s called the *assertive telephone follow-up intervention*. This is where families receive 1 telephone call within 72 h of discharge from the emergency department, and then weekly calls until the young person has attended two scheduled outpatient treatment appointments in a row. These researchers suggest that calls include: brief mood check and suicide risk assessment, safety plan review and revision, reiteration of the importance of lethal means restriction, reiteration of the plan for outpatient treatment developed in the emergency department, enhancing treatment motivation through problem solving of any obstacles to treatment, and providing additional referrals as needed.

### Caring contacts

Another group of researchers have developed and implemented what is called the *caring contacts intervention*. This is where the young person receives caring and hopeful letters, postcards or text messages from the clinician they saw in the emergency department at set intervals (usually no more often than weekly) for a period of time after discharge from the emergency department. An example message is “It has been some time since you were here at the hospital, and we hope things are going well for you. If you wish to drop us a note we would be glad to hear from you”.
